# Increased Mortality in a Nationwide Study of Gastrointestinal Hospitalizations in the United States During the 2020 Coronavirus Pandemic

**DOI:** 10.7759/cureus.66931

**Published:** 2024-08-15

**Authors:** Bishoy Lawendy, Ayooluwatomiwa Adekunle, Muni Rubens, Oyedotun Babajide, Mary Sedarous, Tahniyat Tariq, Philip Okafor

**Affiliations:** 1 Department of Internal Medicine, University of Western Ontario, London, CAN; 2 Department of Gastroenterology, Washington University School of Medicine, St. Louis, USA; 3 Office of Clinical Research, Miami Cancer Institute, Miami, USA; 4 Department of Internal Medicine, Interfaith Medical Center, Brooklyn, USA; 5 Department of Gastroenterology, Queen's University, Kingston, CAN; 6 Department of Gastroenterology, Stanford University, Stanford, USA; 7 Division of Gastroenterology and Hepatology, Mayo Clinic, Jacksonville, USA

**Keywords:** shift in admission trends, healthcare outcomes, hospital admission, gastrointestinal disease, covid-19

## Abstract

Background

The impact of the coronavirus disease-2019 (COVID-19) pandemic on patients with acute gastrointestinal (GI) presentations including acute pancreatitis, diverticulitis, and GI bleeding, requiring hospitalization, has not been fully characterized at the population level in the United States.

Aims

We used the National Inpatient Sample to describe inpatient gastroenterology outcomes in the United States during the first year of the pandemic (2020), using 2018 and 2019 as comparator years.

Methods

Using the National Inpatient Sample, we explored year-to-year and month-to-month trends in hospitalizations, length of stay, and inpatient mortality for GI presentations, including luminal, biliary, infectious, inflammatory, and pancreatic diseases, with regression modeling. Relative change was used to compare time periods.

Results

We observed significantly lower rates of hospitalization for most acute GI conditions in 2020 relative to 2019. Despite this, we noted an increase in all-cause mortality (0.9% in 2019 and 1.1% in 2020, p<0.001) and hospital costs for patients hospitalized with acute presentations of GI-related conditions in 2020 relative to 2019. Importantly, we also observed increased mortality among COVID-19-positive patients who were hospitalized for acute pancreatitis (OR 2.56; 95% CI 1.37-6.53), variceal upper GI bleeding (OR 2.88; 95% CI 1.29-3.84), ulcerative colitis (OR 4.50; 95% CI 1.14-7.74), and acute cholangitis (OR 2.43; 95% CI 1.14-4.93). In 2020, the lowest number of admissions for all conditions occurred in April, coinciding with lockdowns ordered by most state governments.

Conclusions

Acute GI-related hospitalizations, in general, decreased in 2020 but this was associated with higher hospital costs and all-cause mortality increased compared with the pre-pandemic period.

## Introduction

Gastrointestinal (GI) illnesses, including luminal and pancreatic diseases, are major contributors to the utilization and expenditure of healthcare services in the United States. In 2014 alone, over three million hospital admissions in the United States were related to GI diseases, resulting in healthcare costs of over 30 billion dollars [1]. Additionally, there were more than 15.7 million GI-related emergency department visits in the same period [1]. By 2018, the burden of adult hospital admissions with a principal GI diagnosis had increased to almost 4 million and this was met with hospital costs of more than $47 billion [2]. Of these GI admissions, GI bleeding accounted for the majority [2].

In December 2019, a novel human coronavirus emerged for the first time in Wuhan City, central Hubei Province, China [3]. The coronavirus disease 2019 (COVID-19) pandemic caused by the SARS-CoV-2 virus has had a widespread impact on the world and was declared a global pandemic by the World Health Organization (WHO) on March 12, 2020 [3]. The pandemic caused significant disruptions in the healthcare system, affecting both the safety and work processes of healthcare personnel and patients [3,4].

The impact of the COVID-19 pandemic on specific GI diseases and from single health centers has been described in the literature. For instance, some studies reported a decrease in endoscopic resource utilization among patients hospitalized with upper GI bleeding that led to longer hospital stays and higher rates of rebleeding and death. Other studies have shown that patients with upper GI bleeding who required hospitalization during the COVID-19 pandemic tended to be associated with a higher incidence of malignancy [4]. Others found no difference in the number of patients with hemodynamically unstable GI bleeding during the pandemic [5]. Single health system studies such as those from the Veterans Health Administration database revealed a decline in hospitalizations caused by cirrhosis during the COVID-19 pandemic [6], while others reported a surge of cirrhosis-related mortality [7]. Another single health system study of patients with acute pancreatitis (AP) during the COVID-19 pandemic reported fewer admissions; however, those who were hospitalized were more likely to present with symptoms of systemic inflammatory response syndrome, pancreatic necrosis, and persistent organ failure than those admitted prior to the pandemic [7,8].​​

There is a paucity of data on the impact of the COVID-19 pandemic on the spectrum of the most prevalent inpatient GI diseases at the national level in the United States. The conclusions of the aforementioned single-center studies may not be necessarily generalizable. To address this knowledge gap, we used the largest national inpatient dataset to compare trends and outcomes of hospitalizations related to acute GI admissions during the pandemic (2020) relative to the pre-pandemic period (2018-2019). We also performed a month-to-month analysis of GI outcomes during the different phases of the COVID-19 pandemic in 2020. This representative nature of the database allows for robust conclusions and identification of the highest-risk patient cohorts during the pandemic.

## Materials and methods

Data source

We utilized data from the National Inpatient Sample (NIS) including the years 2018 to 2020 [9]. The NIS is the largest all-payer administrative database maintained by the US Agency for Healthcare Research and Quality (AHRQ) and contains de-identified patient-level data from hospital discharges in participating states. It includes both demographic and clinical data [9]. 

Study population and variables

The sample comprised adult (≥18 years) hospitalizations for GI disease in the United States between January 1, 2018, and December 31, 2020. Hospitalizations were included if the primary or secondary discharge diagnosis was one of the following: GI bleeding, AP, cholelithiasis, diverticulitis, non-infectious gastroenteritis, *Clostridium difficile*, viral gastroenteritis, ulcerative colitis, or acute cholangitis. These diagnoses were defined using the International Classification of Diseases, 10th Revision-Clinical Modification (ICD-10-CM) codes.

Outcome measures

The primary outcome measures were month-to-month and year-to-year trends in the GI-related number of hospitalizations in 2018, 2019, and 2020. The secondary outcomes included in-hospital all-cause mortality and hospitalization costs. We also compared outcomes for patients with GI-related presentations who were also COVID-19 positive relative to those who were not. We also investigated independent predictors of mortality in these patients, including clinical and demographic factors. 

Statistical analysis

All statistical analyses were performed using SAS (version 9.4; SAS Institute, Cary, NC). Statistical significance was set at p <0.05, and all tests were two-tailed. Descriptive statistics describing demographics, hospital, and clinical characteristics were generated and compared between 2018, 2019, and 2020. Outcome measures, including rates of hospitalization, length of stay, mortality, and costs, were also compared between 2018, 2019, and 2020. Month-to-month trend analysis was used to compare variables monthly. The chi-square test was used to compare categorical variables. Trends over time were assessed using linear and logistic regression analyses. Weighted multivariable logistic regression analyses were used to identify the factors associated with mortality among COVID-19 hospitalizations, after adjusting for covariates. Appropriateness of the study procedures was ensured using the guidelines developed by Khera and Krumholz for NIS data [10]. This study was exempted by the Mayo Clinic Institutional Review Board because it did not meet the definition of human subject research by the Department of Health and Human Services.

## Results

Patient demographics

Nationally, the majority of hospitalizations related to GI diseases occurred in the age group of 65-84 years, accounting for 37% of all GI-related hospitalizations. The lowest number of hospitalizations was reported in the ≥85 years age group, which accounted for only 7.7%. Between 2018 and 2020, the proportion of patients by race remained stable, with the majority of patients being white (67.8%) or female (52.6%) (Table 1). In 2020, Medicare (47.1%) and private pay (27.8%) were the leading payers for hospitalizations, similar to 2018 and 2019. There was no significant difference observed in hospitalization by covariates such as income, hospital size, region, location, and teaching status in 2020 compared with the pre-pandemic years (Table 1). Importantly, in 2020, patients with GI-related admissions had a statistically significant increase in all-cause mortality relative to prior years (0.9% in 2019 and 1.1% in 2020, p<0.001) (Table 1). 

**Table 1:** Yearly trends in demographic and clinical characteristics of hospitalizations with GI conditions in the United States, 2018-2020 GI, gastrointestinal; NA, not applicable.

Variables	2018	2019	2020	p-Value
Age in years, n (%)				<0.001
18-44	323,800 (22.3%)	319,060 (22.0%)	290,485 (22.9%)	
45-64	486,865 (33.6%)	478,075 (32.9%)	412,290 (32.5%)	
65-84	520,100 (35.9%)	535,080 (36.8%)	469,205 (37.0%)	
≥85	119,930 (8.3%)	120,655 (8.3%)	97,790 (7.7%)	
Sex, n (%)				<0.001
Male	686,180 (46.2%)	687,270 (46.3%)	615,360 (47.4%)	
Female	798,540 (53.8%)	798,190 (53.7%)	683,850 (52.6%)	
Race, n (%)				0.977
White	985,965 (67.9%)	993,890 (68.3%)	862,870 (67.8%)	
African American	193,710 (13.3%)	194,895 (13.4%)	175,500 (13.8%)	
Hispanic	187,300 (12.9%)	180,395 (12.4%)	157,800 (12.4%)	
Asian Pacific Islander	35,305 (2.4%)	35,930 (2.5%)	30,955 (2.4%)	
Native American	9,945 (0.7%)	10,470 (0.7%)	9,535 (0.7%)	
Other	40,705 (2.8%)	40,010 (2.7%)	35,110 (2.8%)	
Payer, n (%)				0.022
Medicare	709,615 (47.9%)	715,475 (48.2%)	611,025 (47.1%)	
Medicaid	225,875 (15.2%)	221,080 (14.9%)	205,140 (15.8%)	
Private	418,025 (28.2%)	414,750 (28.0%)	360,980 (27.8%)	
Self-pay	87,825 (5.9%)	88,220 (5.9%)	79,225 (6.1%)	
No charge	7,540 (0.5%)	7,340 (0.5%)	6,810 (0.5%)	
Other	34,035 (2.3%)	36,985 (2.5%)	34,390 (2.7%)	
Income quartiles, n (%)				0.074
First quartile	416,975 (28.5%)	433,345 (29.6%)	375,320 (29.3%)	
Second quartile	396,115 (27.1%)	371,195 (25.4%)	348,080 (27.2%)	
Third quartile	354,910 (24.3%)	361,770 (24.7%)	299,540 (23.4%)	
Fourth quartile	292,745 (20.0%)	295,595 (20.2%)	256,370 (20.0%)	
Hospital size, n (%)				0.664
Small	337,954 (22.8%)	357,284 (24.1%)	318,395 (24.5%)	
Medium	455,260 (30.7%)	449,075 (30.2%)	379,970 (29.2%)	
Large	691,581 (46.6%)	679,171 (45.7%)	600,901 (46.2%)	
Hospital region, n (%)				0.999
Northeast	278,265 (18.7%)	275,511 (18.5%)	238,140 (18.3%)	
Midwest	320,860 (21.6%)	318,505 (21.4%)	281,475 (21.7%)	
South	589,121 (39.7%)	596,475 (40.2%)	520,690 (40.1%)	
West	296,549 (20.0%)	295,040 (19.9%)	258,960 (19.9%)	
Hospital location and teaching status, n (%)				0.023
Rural	139,464 (9.4%)	133,990 (9.0%)	117,590 (9.1%)	
Urban nonteaching	346,646 (23.3%)	306,625 (20.6%)	263,615 (20.3%)	
Urban teaching	998,685 (67.3%)	1,044,915 (70.3%)	918,059 (70.7%)	
Elixhauser comorbidity index, n (%)				<0.001
0	145,320 (9.8%)	138,100 (9.3%)	109,440 (8.4%)	
1	233,590 (15.7%)	225,590 (15.2%)	184,385 (14.2%)	
2	276,585 (18.6%)	272,605 (18.4%)	230,130 (17.7%)	
≥3	829,300 (55.9%)	849,235 (57.2%)	775,310 (59.7%)	
All-cause mortality, n (%)	13,380 (0.9%)	13,460 (0.9%)	13,835 (1.1%)	<0.001
COVID-19, n (%)	NA	NA	17,995 (1.4%)	NA
COVID-19 mortality, n (%)	NA	NA	960 (5.3%)	NA
Viral pneumonia, n (%)	420	480	6,660 (0.5%)	<0.001
Viral pneumonia mortality, n (%)	11 (2.4%)	20 (4.2%)	785 (11.9%)	0.002

Year-to-year national hospitalization trends 

Compared to 2019, our results showed that there was a significant decrease in hospitalizations in 2020 for AP (relative change [RC] negative 7%, p<0.001), cholelithiasis (RC -14.9%, p<0.001), diverticulitis (RC -17.3%, p<0.001), non-infectious colitis (RC -22.4, p<0.001), nonvariceal GI bleeding (RC -7.5%, p 0.002), and viral gastroenteritis (RC -32.3%, p<0.001). For viral gastroenteritis, the number of discharges decreased from 75,300 in 2019 to 51,765 in 2020 (p<0.001). There was no significant difference in the rate of hospitalization for variceal upper GI bleeding and lower GI bleeding (including diverticular bleeding), *Clostridium difficile*, ulcerative colitis, or acute cholangitis. We observed a significant increase in mortality among patients with ulcerative colitis (RC 25.9%, p=0.007) and noninfectious gastroenteritis/colitis (RC 55.6%, p=0.023) in 2020 relative to 2019. Relative to 2019, we observed a significant increase in hospitalization costs in 2020 under all investigated conditions. The length of hospital stay and hospital cost data are summarized in Table 2. 

**Table 2:** Yearly trends in number of discharges, length of stay, hospitalization cost, and mortality rate of selected GI condition discharges in the United States, 2018-2020 LOS, length of stay; GI, gastrointestinal.

Liver conditions	2018	2019	2020	Relative change (%)	p-Value for trend
Acute pancreatitis					
Total number of discharges, n (%)	278,365 (0.9%)	275,145 (0.9%)	258,965 (0.9%)	-7	<0.001
LOS, median (IQR)	2.7 (1.5-4.3)	2.7 (1.5-4.3)	2.7 (1.5-4.4)	0	0.648
Total hospital stays (in thousands) days	1,175	1,154	1,098	-6.6	
Hospital costs, median (IQR)	6,518 (4,373-10,664)	6,911 (4,583-11,248)	7,421 (4,908-12,245)	13.9	<0.001
Mortality, n (%)	1,500 (0.2%)	1,600 (0.2%)	1,600 (0.2%)	6.7	0.242
Cholelithiasis					
Total number of discharges, n (%)	263,835 (0.9%)	260,530 (0.9%)	224,410 (0.8%)	-14.9	<0.001
LOS, median (IQR)	2.4 (1.3-3.9)	2.4 (1.3-3.9)	2.4 (1.2-3.9)	0.0	0.529
Total hospital stays (in thousands) days	953.0	951.0	815.0	-14.5	
Hospital costs, median (IQR)	11,034 (7,818-15,647)	11,695 (8,193-16,629)	12,655 (8,909-17,949)	14.7	<0.001
Mortality, n (%)	930 (0.1%)	965 (0.1%)	790 (0.1%)	-15.1	0.490
Diverticulitis					
Total number of discharges	209,475 (0.7%)	210,875 (0.7%)	173,195 (0.6%)	-17.3	<0.001
LOS, median (IQR)	2.8 (1.7-4.7)	2.8 (1.7-4.7)	2.8 (1.7-4.8)	0	0.398
Total hospital stays (in thousands) days	941	947	783	-16.8	
Hospital costs, median (IQR)	7,182 (4,536-14,106)	7,545 (4,756-15,027)	8,411 (5,138-16,769)	17.1	<0.001
Mortality, n (%)	780 (0.1%)	830 (0.1%)	945 (0.1%)	21.2	0.123
Noninfectious gastroenteritis/colitis					
Total number of discharges	100,225 (0.3%)	98,995 (0.3%)	77,795 (0.3%)	-22.4	<0.001
LOS, median (IQR)	2.0 (1.1-3.5)	2.1 (1.1-3.5)	2.1 (1.2-3.6)	5	<0.001
Total hospital stays (in thousands) days	319	325	261	-18.2	
Hospital costs, median (IQR)	5,709 (4,073-8,250)	6,007 (4,251-8,865)	6,578 (4,625-9,681)	15.2	<0.001
Mortality, n (%)	270 (0.0%)	485 (0.1%)	420 (0.0%)	55.6	0.023
Nonvariceal upper GI bleeding					
Total number of discharges	294,225 (1.0%)	298,485 (1.0%)	272,285 (1.0%)	-7.5	0.002
LOS, median (IQR)	2.8 (1.6-4.6)	2.8 (1.6-4.7)	2.8 (1.7-4.8)	0	<0.001
Total hospital stays (in thousands) days	1,262	1,324	1,221	-3.2	
Hospital costs, median (IQR)	8,372 (5,741-12,852)	8,926 (6,090-13,776)	9,611 (6,546-14,956)	14.8	<0.001
Mortality, n (%)	5,450 (0.8%)	5,145 (0.8%)	5,255 (0.6%)	-3.6	0.105
Variceal upper GI bleeding					
Total number of discharges	7,370 (0.0%)	7,215 (0.0%)	6,305 (0.0%)	-14.5	0.906
LOS, median (IQR)	3.1 (2.0-4.8)	3.1 (1.9-4.8)	3.1 (2.0-4.8)	0	0.476
Total hospital stays (in thousands) days	34	33	29	-14.7	
Hospital costs, median (IQR)	10,546 (7,228-16,826)	10,898 (7,672-16,554)	11,700 (7,975-17,984)	10.9	0.023
Mortality, n (%)	400 (0.1%)	355 (0.1%)	340 (0.0%)	-15	0.560
Lower GI bleeding and diverticular bleeding					
Total number of discharges	127,305 (0.4%)	131,750 (0.4%)	118,830 (0.4%)	-6.7	0.288
LOS, median (IQR)	2.7 (1.5-4.4)	2.8 (1.6-4.6)	2.8 (1.6-4.7)	3.7	<0.001
Total hospital stays (in thousands) days	520	556	517	-0.6	
Hospital costs, median (IQR)	7,318 (4,815-11,590)	7,950 (5,233-12,721)	8,807 (5,784-14,084)	20.3	<0.001
Mortality, n (%)	1,275 (0.2%)	1,225 (0.2%)	1,275 (0.1%)	0	0.744
Clostridium difficile					
Total number of discharges	18,725 (0.1%)	16,890 (0.1%)	11,470 (0.0%)	-38.7	0.73
LOS, median (IQR)	3.9 (2.4-6.4)	4.0 (2.4-6.6)	3.9 (2.4-6.8)	0	0.29
Total hospital stays (in thousands) days	110	100	69	-37.3	
Hospital costs, median (IQR)	7,233 (4,769-11,606)	7,870 (5,280-12,536)	8,841 (5,891-14,394)	22.2	<0.001
Mortality, n (%)	200 (0.0%)	250 (0.0%)	120 (0.0%)	-40	0.81
Viral gastroenteritis					
Total number of discharges	76,460 (0.3%)	75,300 (0.2%)	51,765 (0.2%)	-32.3	<0.001
LOS, median (IQR)	2.0 (1.1-3.4)	2.1 (1.2-3.4)	2.2 (1.2-3.6)	10	<0.001
Total hospital stays (in thousands) days	239	241	174	-27.2	
Hospital costs, median (IQR)	5,576 (3,986-8,049)	5,902 (4,264-8,533)	6,606 (4,702-9,500)	18.5	<0.001
Mortality, n (%)	175 (0.0%)	245 (0.0%)	160 (0.0%)	-8.6	0.80
Ulcerative colitis					
Total number of discharges	123,140 (0.4%)	126,845 (0.4%)	118,565 (0.4%)	-3.7	0.076
LOS, median (IQR)	3.2 (1.8-5.9)	3.2 (1.7-5.9)	3.4 (1.8-6.2)	6.2	0.027
Total hospital stays (in thousands) days	693	701	688	-0.7	
Hospital costs, median (IQR)	8,979 (5,459-16,231)	9,551 (5,762-17,356)	10,626 (6,316-19,497)	18.3	<0.001
Mortality, n (%)	2,475 (0.4%)	2,495 (0.4%)	3,115 (0.4%)	25.9	0.007
Acute cholangitis					
Total number of discharges	14,790 (0.0%)	13,445 (0.0%)	12,740 (0.0%)	-13.9	0.134
LOS, median (IQR)	2.9 (1.8-4.7)	2.8 (1.7-4.8)	2.8 (1.7-4.5)	-3.4	0.045
Total hospital stays (in thousands) days	67	60	54	-19.4	
Hospital costs, median (IQR)	10,442 (7,194-15,478)	11,021 (7,491-16,518)	11,711 (7,736-17,540)	12.2	0.007
Mortality, n (%)	130 (0.0%)	160 (0.0%)	90 (0.0%)	-30.8	0.943

Month-to-month national hospitalization trends

In general, most admissions decreased in 2020 relative to their analogous months in 2019, except for January 2020. January 2020 had more admissions than January 2019 for the following: AP, diverticulitis, variceal and nonvariceal upper GI bleeding, and lower GI bleeding. The peak number of admissions for most conditions (AP, cholelithiasis, diverticulitis, noninfectious gastroenteritis/colitis, nonvariceal upper GI bleeding, lower GI bleeding, *Clostridium difficile*, ulcerative colitis, and viral gastroenteritis) occurred in January 2020 which likely still represents the pre-pandemic era. The lowest number of admissions for all conditions occurred in April 2020 which corresponds to the initial phase of government lockdowns. In 2019, the lowest number of admissions for most conditions (AP, diverticulitis, noninfectious gastroenteritis/colitis, nonvariceal upper GI bleeding, lower GI bleeding, and ulcerative colitis) was during the month of February. In terms of mortality trends, for diverticulitis, mortality was higher in 2020 than in 2019 for all months except June, August, October, and November. For non-variceal upper GI bleeding, mortality was higher in 2020 than in 2019 for all months, except January and February. 

For noninfectious gastroenteritis, mortality was similar between 2019 and 2020 for most of the year although there was a higher mortality in January, May, July, and December. For AP, 2020 had higher mortality in April, May, September, October, November, and December, whereas 2019 had higher mortality in March, June, and July. For ulcerative colitis, mortality was higher in 2020 than in 2019 for all months except June, September, and October. The peak mortality for most GI conditions in 2020 occurred in the months of November and December including AP, cholelithiasis, diverticulitis, noninfectious gastroenteritis/colitis, nonvariceal upper GI bleeding, lower GI bleeding, diverticular bleeding, and ulcerative colitis. Full mortality trends month by month are illustrated in Tables 3, 4.

**Table 3:** Monthly trends in the number of discharges and mortality rate of selected GI condition discharges in the United States, 2020 GI, gastrointestinal.

	January	February	March	April	May	June	July	August	September	October	November	December	Relative change (%)	p-Value for trend
	2020	2020	2020	2020	2020	2020	2020	2020	2020	2020	2020	2020		
Acute pancreatitis														
Total number of discharges, n (%)	23,560 (0.9%)	21,085 (0.9%)	19,575 (0.9%)	17,810 (1.0%)	22,075 (1.1%)	23,300 (1.0%)	23,130 (1.0%)	23,005 (1.0%)	22,910 (1.0%)	22,005 (0.9%)	20,060 (0.9%)	20,225 (0.8%)	-11.1	0.038
Mortality, n (%)	115 (0.5%)	105 (0.5%)	120 (0.6%)	125 (0.7%)	155 (0.7%)	85 (0.4%)	140 (0.6%)	140 (0.6%)	200 (0.9%)	130 (0.6%)	140 (0.7%)	140 (0.7%)	40	0.085
Cholelithiasis														
Total number of discharges, n (%)	21,260 (0.8%)	20,050 (0.8%)	16,050 (0.7%)	13,800 (0.8%)	18,030 (0.9%)	19,375 (0.8%)	19,395 (0.8%)	19,725 (0.8%)	19,785 (0.9%)	20,935 (0.9%)	17,635 (0.8%)	18,240 (0.8%)	0	0.935
Mortality, n (%)	65 (0.3%)	85 (0.4%)	45 (0.3%)	40 (0.3%)	50 (0.3%)	55 (0.3%)	40 (0.2%)	95 (0.5%)	65 (0.3%)	85 (0.4%)	75 (0.4%)	90 (0.5%)	66.7	0.131
Diverticulitis														
Total number of discharges, n (%)	17,090 (0.7%)	16,570 (0.7%)	12,945 (0.6%)	8,725 (0.5%)	12,325 (0.6%)	15,100 (0.7%)	15,000 (0.6%)	15,715 (0.7%)	15,875 (0.7%)	16,085 (0.7%)	13,825 (0.6%)	13,875 (0.6%)	-14.3	0.665
Mortality, n (%)	105 (0.6%)	100 (0.6%)	100 (0.8%)	50 (0.6%)	90 (0.7%)	55 (0.4%)	75 (0.5%)	65 (0.4%)	60 (0.4%)	60 (0.4%)	75 (0.5%)	110 (0.8%)	33.3	0.302
Noninfectious gastroenteritis/colitis														
Total number of discharges, n (%)	8,090 (0.3%)	7,415 (0.3%)	6,350 (0.3%)	4,505 (0.3%)	6,095 (0.3%)	6,430 (0.3%)	7,020 (0.3%)	6,725 (0.3%)	6,980 (0.3%)	6,755 (0.3%)	5,935 (0.3%)	5,345 (0.2%)	-33.3	<0.001
Mortality, n (%)	65 (0.8%)	25 (0.3%)	45 (0.7%)	25 (0.6%)	55 (0.9%)	20 (0.3%)	25 (0.4%)	20 (0.3%)	35 (0.5%)	25 (0.4%)	20 (0.3%)	60 (1.1%)	37.5	0.638
Nonvariceal upper GI bleeding														
Total number of discharges, n (%)	25,890 (1.0%)	24,295 (1.0%)	21,970 (1.0%)	18,330 (1.0%)	22,075 (1.1%)	22,990 (1.0%)	23,325 (1.0%)	23,050 (1.0%)	23,420 (1.0%)	23,595 (1.0%)	21,470 (0.9%)	21,715 (0.9%)	-10	<0.001
Mortality, n (%)	390 (1.5%)	405 (1.7%)	400 (1.8%)	425 (2.3%)	430 (1.9%)	430 (1.9%)	515 (2.2%)	380 (1.6%)	435 (1.9%)	490 (2.1%)	490 (2.3%)	460 (2.1%)	40	0.014
Variceal upper GI bleeding														
Total number of discharges, n (%)	605 (0.0%)	545 (0.0%)	595 (0.0%)	345 (0.0%)	485 (0.0%)	570 (0.0%)	415 (0.0%)	535 (0.0%)	560 (0.0%)	670 (0.0%)	435 (0.0%)	535 (0.0%)	0	0.660
Mortality, n (%)	15 (2.5%)	20 (3.7%)	25 (4.2%)	25 (7.2%)	45 (9.3%)	25 (4.4%)	35 (8.4%)	50 (9.4%)	50 (8.9%)	20 (3.0%)	20 (4.6%)	10 (1.9%)	-24	0.805
Lower GI bleeding and diverticular bleeding														
Total number of discharges, n (%)	11,485 (0.4%)	11,015 (0.5%)	9,740 (0.4%)	7,620 (0.4%)	9,295 (0.4%)	9,390 (0.4%)	9,995 (0.4%)	9,855 (0.4%)	10,110 (0.4%)	10,505 (0.4%)	9,670 (0.4%)	10,075 (0.4%)	0	0.535
Mortality, n (%)	80 (0.7%)	85 (0.8%)	140 (1.4%)	90 (1.2%)	95 (1.0%)	75 (0.8%)	95 (1.0%)	115 (1.2%)	100 (1.0%)	100 (1.0%)	115 (1.2%)	180 (1.8%)	157.1	0.020
Clostridium difficile														
Total number of discharges, n (%)	1,270 (0.0%)	1,205 (0.1%)	975 (0.0%)	695 (0.0%)	880 (0.0%)	810 (0.0%)	915 (0.0%)	1110 (0.0%)	980 (0.0%)	990 (0.0%)	770 (0.0%)	850 (0.0%)	0	0.525
Mortality, n (%)	10 (0.8%)	5 (0.4%)	25 (2.6%)	5 (0.7%)	10 (1.1%)	10 (1.2%)	15 (1.6%)	15 (1.4%)	0	15 (1.5%)	5 (0.6%)	5 (0.6%)	-25	0.706
Viral gastroenteritis														
Total number of discharges, n (%)	5,950 (0.2%)	5,390 (0.2%)	4,600 (0.2%)	2,985 (0.2%)	3,690 (0.2%)	4,450 (0.2%)	4,375 (0.2%)	4,580 (0.2%)	4,450 (0.2%)	4,250 (0.2%)	3,575 (0.2%)	3,355 (0.1%)	-50	<0.001
Mortality, n (%)	20 (0.3%)	5 (0.1%)	5 (0.1%)	15 (0.5%)	10 (0.3%)	10 (0.2%)	5 (0.1%)	35 (0.8%)	20 (0.4%)	15 (0.4%)	10 (0.3%)	10 (0.3%)	0	0.274
Ulcerative colitis														
Total number of discharges, n (%)	10,695 (0.4%)	9,780 (0.4%)	9,205 (0.4%)	7,195 (0.4%)	9,090 (0.4%)	9,695 (0.4%)	10,100 (0.4%)	10,420 (0.4%)	10,535 (0.5%)	11,105 (0.5%)	10,510 (0.5%)	10,000 (0.4%)	0	0.442
Mortality, n (%)	295 (2.8%)	235 (2.4%)	260 (2.8%)	230 (3.2%)	265 (2.9%)	210 (2.2%)	205 (2.0%)	230 (2.2%)	215 (2.0%)	230 (2.1%)	380 (3.6%)	325 (3.3%)	17.9	0.663
Acute cholangitis														
Total number of discharges, n (%)	995 (0.0%)	1,105 (0.0%)	950 (0.0%)	930 (0.1%)	1,030 (0.0%)	1,105 (0.0%)	1,105 (0.0%)	1,235 (0.1%)	1,135 (0.0%)	1,210 (0.1%)	965 (0.0%)	970 (0.0%)	0	0.517
Mortality, n (%)	0	5 (0.5%)	0	10 (1.1%)	5 (0.5%)	10 (0.9%)	5 (0.5%)	10 (0.8%)	10 (0.9%)	10 (0.8%)	10 (1.0%)	10 (1.0%)	NA	NA

**Table 4:** Monthly trends in the number of discharges and mortality rate of selected GI condition discharges in the United States, 2019 GI, gastrointestinal.

	January	February	March	April	May	June	July	August	September	October	November	December	Relative change (%)	p-Value for trend
	2019	2019	2019	2019	2019	2019	2019	2019	2019	2019	2019	2019		
Acute pancreatitis														
Total number of discharges, n (%)	23,240 (0.9%)	19,670 (0.8%)	22,710 (0.9%)	22,770 (0.9%)	23,280 (0.9%)	23,165 (0.9%)	24,710 (1.0%)	24,260 (1.0%)	23,060 (0.9%)	23,645 (0.9%)	21,855 (0.9%)	22,510 (0.9%)	0	0.501
Mortality, n (%)	115 (0.5%)	100 (0.5%)	150 (0.7%)	135 (0.6%)	125 (0.5%)	130 (0.6%)	185 (0.7%)	150 (0.6%)	135 (0.6%)	105 (0.4%)	125 (0.6%)	145 (0.6%)	20	0.705
Cholelithiasis														
Total number of discharges, n (%)	22,155 (0.8%)	20,490 (0.9%)	21,855 (0.8%)	21,320 (0.8%)	22,455 (0.9%)	21,695 (0.9%)	22,820 (0.9%)	22,260 (0.9%)	22,040 (0.9%)	22,305 (0.9%)	20,600 (0.9%)	20,380 (0.8%)	0	0.799
Mortality, n (%)	55 (0.2%)	85 (0.4%)	60 (0.3%)	55 (0.3%)	65 (0.3%)	95 (0.4%)	140 (0.6%)	45 (0.2%)	105 (0.5%)	65 (0.3%)	100 (0.5%)	95 (0.5%)	150	0.061
Diverticulitis														
Total number of discharges, n (%)	16,990 (0.6%)	15,515 (0.7%)	17,490 (0.7%)	17,590 (0.7%)	18,220 (0.7%)	17,415 (0.7%)	18,255 (0.7%)	19,015 (0.7%)	17,775 (0.7%)	18,950 (0.7%)	16,475 (0.7%)	17,095 (0.7%)	16.7	<0.001
Mortality, n (%)	65 (0.4%)	60 (0.4%)	55 (0.3%)	80 (0.5%)	70 (0.4%)	80 (0.5%)	55 (0.3%)	80 (0.4%)	45 (0.3%)	90 (0.5%)	95 (0.6%)	55 (0.3%)	-25	0.672
Noninfectious gastroenteritis/colitis														
Total number of discharges, n (%)	8,130 (0.3%)	7,460 (0.3%)	8,400 (0.3%)	8,390 (0.3%)	8,580 (0.3%)	8,065 (0.3%)	8,980 (0.4%)	8,575 (0.3%)	8,135 (0.3%)	7,765 (0.3%)	7,840 (0.3%)	8,435 (0.3%)	0	0.259
Mortality, n (%)	45 (0.6%)	25 (0.3%)	55 (0.7%)	50 (0.6%)	65 (0.8%)	25 (0.3%)	20 (0.2%)	30 (0.3%)	40 (0.5%)	30 (0.4%)	50 (0.6%)	50 (0.6%)	0	0.849
Nonvariceal upper GI bleeding														
Total number of discharges, n (%)	25,380 (1.0%)	23,420 (1.0%)	26,310 (1.0%)	25,090 (1.0%)	25,890 (1.0%)	24,190 (1.0%)	25,365 (1.0%)	24,960 (1.0%)	23,575 (1.0%)	25,455 (1.0%)	24,425 (1.0%)	24,225 (1.0%)	0	0.326
Mortality, n (%)	515 (2.0%)	450 (1.9%)	350 (1.3%)	400 (1.6%)	445 (1.7%)	390 (1.6%)	405 (1.6%)	345 (1.4%)	425 (1.8%)	495 (1.9%)	455 (1.9%)	470 (1.9%)	-5	0.506
Variceal upper GI bleeding														
Total number of discharges, n (%)	595 (0.0%)	565 (0.0%)	675 (0.0%)	620 (0.0%)	580 (0.0%)	570 (0.0%)	560 (0.0%)	545 (0.0%)	610 (0.0%)	650 (0.0%)	595 (0.0%)	650 (0.0%)	0	0.546
Mortality, n (%)	35 (5.9%)	25 (4.4%)	35 (5.2%)	25 (4.0%)	20 (3.4%)	40 (7.0%)	30 (5.4%)	15 (2.8%)	40 (6.6%)	25 (3.8%)	30 (5.0%)	35 (5.4%)	-8.5	0.996
Lower GI bleeding and diverticular bleeding														
Total number of discharges, n (%)	11,155 (0.4%)	10,235 (0.4%)	11,665 (0.5%)	11,280 (0.4%)	11,555 (0.4%)	10,585 (0.4%)	10,980 (0.4%)	10,700 (0.4%)	10,570 (0.4%)	11,335 (0.4%)	10,645 (0.4%)	11,000 (0.4%)	0	0.807
Mortality, n (%)	150 (1.3%)	45 (0.4%)	85 (0.7%)	100 (0.9%)	130 (1.1%)	100 (0.9%)	110 (1.0%)	120 (1.1%)	85 (0.8%)	145 (1.3%)	90 (0.8%)	65 (0.6%)	-53.8	0.781
Clostridium difficile														
Total number of discharges, n (%)	1,460 (0.1%)	1,310 (0.1%)	1,415 (0.1%)	1,290 (0.1%)	1,480 (0.1%)	1,465 (0.1%)	1,450 (0.1%)	1,370 (0.1%)	1,505 (0.1%)	1,375 (0.1%)	1,375 (0.1%)	1,390 (0.1%)	0	0.666
Mortality, n (%)	25 (1.7%)	15 (1.1%)	25 (1.8%)	20 (1.6%)	15 (1.0%)	15 (1.0%)	20 (1.4%)	25 (1.8%)	35 (2.3%)	10 (0.7%)	15 (1.1%)	30 (2.2%)	29.4	0.820
Viral gastroenteritis														
Total number of discharges, n (%)	6,690 (0.3%)	5,775 (0.2%)	6,905 (0.3%)	6,860 (0.3%)	6,300 (0.2%)	6,045 (0.2%)	6,475 (0.3%)	6,265 (0.2%)	6,050 (0.2%)	5,635 (0.2%)	5,660 (0.2%)	6,470 (0.3%)	0	0.324
Mortality, n (%)	10 (0.1%)	15 (0.3%)	30 (0.4%)	20 (0.3%)	15 (0.2%)	15 (0.2%)	20 (0.3%)	15 (0.2%)	30 (0.5%)	25 (0.4%)	25 (0.4%)	25 (0.4%)	300	0.175
Ulcerative colitis														
Total number of discharges, n (%)	10,840 (0.4%)	9,575 (0.4%)	10,615 (0.4%)	10,455 (0.4%)	10,730 (0.4%)	10,140 (0.4%)	10,845 (0.4%)	11,020 (0.4%)	10,925 (0.4%)	10,845 (0.4%)	10,360 (0.4%)	10,365 (0.4%)	0	0.230
Mortality, n (%)	140 (1.3%)	175 (1.8%)	190 (1.8%)	140 (1.3%)	230 (2.1%)	250 (2.5%)	205 (1.9%)	210 (1.9%)	215 (2.0%)	230 (2.1%)	260 (2.5%)	250 (2.4%)	84.6	0.002
Acute cholangitis														
Total number of discharges, n (%)	1,055 (0.0%)	1,045 (0.0%)	1,070 (0.0%)	985 (0.0%)	1,165 (0.0%)	1,170 (0.0%)	1,295 (0.1%)	1,200 (0.0%)	1,090 (0.0%)	1,055 (0.0%)	1,130 (0.0%)	1,160 (0.0%)	0	0.356
Mortality, n (%)	10 (0.9%)	5 (0.5%)	30 (2.8%)	5 (0.5%)	15 (1.3%)	5 (0.4%)	15 (1.2%)	5 (0.4%)	10 (0.9%)	20 (1.9%)	15 (1.3%)	25 (2.2%)	144.4	0.406

COVID-19-positive hospitalizations for GI conditions and outcomes 

We also explored the influence of COVID-19 on mortality in patients with GI presentations. It is important to note that the COVID-19 ICD-10 code was introduced in March 2020 [11]. This raises the possibility of misclassification, as COVID-19 presentations would most likely be coded differently. To mitigate this, we also reported viral pneumonia admissions in patients with GI-related presentations. We also explored factors associated with mortality among COVID-positive GI conditions (Table 5). As a general trend, the odds ratio for mortality increased with age. Patients aged 65-74 and ≥75 years had significantly increased mortality (OR 3.56, CI (1.35-9.40), p=0.0086) and (OR 4.69, CI (2.68-6.38), p<0.001), respectively. Furthermore, women were less likely to die (OR 0.63, CI (0.45-0.90), p=0.0106) compared to men. There were no statistically significant effects of race, primary payer, or income on mortality in our patient sample. Native Americans had the highest OR for mortality; however, this trend was not statistically significant (OR 1.34, CI (0.26-6.86), p=0.5162). Patients in large hospitals and urban teaching hospitals had higher mortality (OR 2.18, CI (1.36-3.50), p<0.001) and (OR 2.11, CI (1.04-4.28), p=0.0277), respectively, though hospital region had no impact on mortality. 

**Table 5:** Factors associated with mortality among COVID-positive patients presenting with GI conditions CRF, chronic renal failure; GI, gastrointestinal.

Variables	OR (95% CI)	p-Value
Age group		
18-39	Reference	
40-64	2.05 (1.02-3.12)	0.059
65-74	3.56 (1.35-9.40)	0.009
≥75	4.69 (2.68-6.38)	<0.001
Sex		
Male	Reference	
Female	0.63 (0.45-0.90)	0.011
Race		
White	Reference	
African American	0.78 (0.46-1.32)	0.756
Hispanic	1.02 (0.60-1.73)	0.517
Asian Pacific Islander	0.58 (0.13-2.58)	0.557
Native American	1.34 (0.26-6.86)	0.516
Other/missing	0.62 (0.21-1.80)	0.507
Primary payer		
Medicare	Reference	
Medicaid	0.72 (0.29-1.79)	0.423
Private	1.10 (0.61-1.99)	0.323
Self-pay	2.07 (0.71-4.02)	0.431
No charge	0.84 (0.56-1.63)	0.253
Other	1.15 (0.43-3.08)	0.533
Income		
Q1	Reference	
Q2	0.60 (0.38-1.93)	0.300
Q3	0.65 (0.40-1.05)	0.650
Q4	0.61 (0.37-1.02)	0.442
Hospital size		
Small	Reference	
Medium	1.41 (0.82-2.42)	0.815
Large	2.18 (1.36-3.50)	<0.001
Hospital region		
Northeast	Reference	
Midwest	0.57 (0.36-0.90)	0.233
South	0.64 (0.41-1.00)	0.675
West	0.58 (0.33-1.01)	0.394
Hospital location and teaching status		
Rural	Reference	
Urban nonteaching	1.64 (0.75-3.59)	0.645
Urban teaching	2.11 (1.04-4.28)	0.028
Elixhauser comorbidity index		
0	Reference	
1	1.00 (0.18-5.60)	0.12
2	2.17 (0.46-10.27)	0.66
≥3	6.01 (1.35-26.75)	<0.0001
Diabetes	1.96 (1.64-2.43)	<0.0002
Hypertension	1.64 (1.42-1.97)	<0.0003
Obesity	1.68 (1.43-2.08)	<0.0004
CRF	1.52 (0.99-2.34)	0.058
Acute pancreatitis	2.56 (1.37-6.53)	0.007
Cholelithiasis	0.67 (0.13-3.47)	0.633
Diverticulitis	1.45 (0.37-5.66)	0.589
Noninfectious gastroenteritis/colitis	0.27 (0.03-2.72)	0.265
Nonvariceal upper GI bleeding	1.26 (0.32-4.93)	0.744
Variceal upper GI bleeding	2.88 (1.29-3.84)	0.017
Lower GI bleeding and diverticular bleeding	0.77 (0.22-2.72)	0.688
Clostridium difficile	3.16 (0.36-7.43)	0.297
Viral gastroenteritis	0.87 (0.65-1.76)	0.214
Ulcerative colitis	4.50 (1.14-7.74)	0.032
Acute cholangitis	2.43 (1.14-4.93)	0.017

We were uniquely able to study other independent predictors of COVID-19 mortality in patients with GI conditions and found that the Elixhauser comorbidity index ≥3 (OR 6.01, CI (1.35-26.75), p<0.0001), the coexistence of diabetes mellitus (OR 1.96, CI (1.64-2.43), p<0.0002), hypertension (OR 1.64, CI (1.42-1.97), p<0.0003), and obesity (OR 1.68, CI (1.43-2.08), p<0.0004) increased COVID-19 mortality among the cohort admitted with GI diseases. We also observed increased mortality in patients who were hospitalized for AP (OR 2.56, CI (1.37-6.53), p=0.007), variceal upper GI bleeding (OR 2.88, CI (1.29-3.84), p=0.017), ulcerative colitis (OR 4.50, CI (1.14-7.74), p=0.0315), and acute cholangitis (OR 2.43, CI (1.14-4.93), p=0.0172) who were also COVID-19 positive. Moreover, we observed an increase in admissions for viral pneumonia in patients presenting with GI-related conditions (420 in 2018, 480 in 2019, and 6660 in 2020; p<0.001), suggesting that indeed some of these cases might have represented COVID-19. This was observed in conjunction with an excess mortality of 7.7% in 2020 relative to 2019 for viral pneumonia (4.2% in 2019 and 11.9% in 2020; p<0.002). 

Sensitivity analysis

To identify potential modifiers of our observations, we performed a sensitivity analysis using interaction terms including time. We have included the results in Table 6. Results were mostly comparable with the original analysis in terms of direction of significance. However, adjusted mortality of ulcerative colitis became nonsignificant in patients with COVID-19.

**Table 6:** Sensitivity analysis using interaction terms including time for factors associated with mortality among COVID-positive patients presenting with GI conditions CRF, chronic renal failure; GI, gastrointestinal.

Variables	OR (95% CI)	p-Value
Year		
2018	Reference	
2019	0.84 (0.23-1.83)	0.526
2020	1.02 (0.76-2.46)	0.372
Age groups		
18-39	Reference	
40-64	1.18 (0.89-3.01)	0.087
65-74	3.75 (1.22-8.32)	<0.001
≥75	4.1 (2.10-5.18)	<0.001
Sex		
Male	Reference	
Female	0.71 (0.70-0.73)	<0.001
Race		
White	Reference	
African American	0.98 (0.94-1.12)	0.252
Hispanic	1.15 (0.87-1.31)	0.334
Asian Pacific Islander	0.64 (9.26-1.98)	0.536
Native American	1.76 (0.53-2.02)	0.534
Other/missing	0.76 (0.35-1.98)	0.249
Primary payer		
Medicare	Reference	
Medicaid	0.63 (0.32-1.63)	0.827
Private	1.03 (0.98-1.28)	0.652
Self-pay	1.29 (0.17-2.42)	0.735
No charge	0.89 (0.61-1.31)	0.635
Other	1.17 (0.54-1.82)	0.536
Income		
Q1	Reference	
Q2	0.53 (0.45-2.14)	0.342
Q3	0.83 (0.79-1.28)	0.453
Q4	0.81 (0.76-1.52)	0.536
Hospital size		
Small	Reference	
Medium	1.16 (0.45-1.22)	0.736
Large	1.18 (1.12-1.24)	<0.001
Hospital region		
Northeast	Reference	
Midwest	0.67 (0.32-1.72)	0.325
South	0.74 (0.59-1.79)	0.273
West	0.81 (0.65-1.86)	0.645
Hospital location and teaching status		
Rural	Reference	
Urban nonteaching	1.34 (0.21-1.4)	0.736
Urban teaching	1.44 (1.36-1.54)	<0.001
Elixhauser comorbidity index		
0	Reference	
1	0.92 (0.82-1.03)	0.241
2	1.14 (0.25-1.97)	0.713
≥3	5.93 (2.62-23.28)	<0.001
Diabetes	1.09 (1.06-1.12)	<0.001
Hypertension	1.67 (1.65-1.69)	<0.001
Obesity	1.04 (1.01-1.08)	0.0042
CRF	1.34 (0.74-1.95)	0.0842
Acute pancreatitis	1.48 (1.44-1.53)	<0.001
Cholelithiasis	1.09 (0.58-2.05)	0.7966
Diverticulitis	1.31 (0.38-4.53)	0.6675
Noninfectious gastroenteritis/colitis	0.64 (0.08-5.38)	0.6781
Nonvariceal upper GI bleeding	1.22 (0.64-1.53)	0.0736
Variceal upper GI bleeding	1.72 (1.53-1.94)	<0.001
Lower GI bleeding and diverticular bleeding	0.71 (0.12-4.3)	0.7082
Clostridium difficile	1.57 (0.37-6.61)	0.5371
Viral gastroenteritis	1.01 (0.48-2.11)	0.9817
Ulcerative colitis	0.57 (0.09-3.79)	0.5639
Acute cholangitis	2.65 (1.28-5.82)	<0.001
Age*Year	0.84 (0.63-1.84)	0.526
Race*Year	0.53 (0.11-1.98)	0.245
Primary payer*Year	1.23 (0.23-2.87)	0.452
Hospital size*Year	1.02 (0.65-1.93)	0.263
Elixhauser comorbidity index*Year	0.71 (0.23-1.98)	0.342

## Discussion

In this population-level study, we evaluated the trends and outcomes of hospitalizations for acute GI conditions in 2020, the first year of the COVID pandemic across the United States, using the NIS. We observed that the demographic and socioeconomic distribution of patients hospitalized in 2020 was comparable with pre-pandemic years. Even though hospitalizations for patients with GI diseases decreased in 2020, all-cause inpatient mortality was significantly higher at 1.1% compared to the pre-pandemic years, when it had remained stable at about 0.9%. We believe this increase in all-cause inpatient mortality is attributable to COVID-19. By performing a month-to-month trend analysis, we found that in 2020, the peak number of admissions for most conditions was during January, while the lowest number of admissions for all conditions was during April, which coincided with lockdowns ordered by most state governments throughout the country [12,13]. 

The decline in hospitalizations during the pandemic is consistent with the published literature in the USA as well as other countries [6,14,15]. The peak mortality for most GI conditions including AP, cholelithiasis, diverticulitis, noninfectious gastroenteritis/colitis, nonvariceal upper GI bleeding, lower GI bleeding and diverticular bleeding, and ulcerative colitis occurred in the months of November and December, which corresponded with the subsequent peaks of the pandemic from novel COVID-19 variants. Our results are in line with other published reports showing increased mortality for hospitalizations in various acute presentations in 2020 [7,16]. Reduced hospitalizations were likely in part driven by the fear of presenting to the hospital and contracting COVID-19, causing delays in care and more acute presentations, leading to higher mortality. This trend has been demonstrated in other disease states such as cirrhosis, heart failure, and myocardial infarctions [7,16-18]. The excess mortality observed in our study is comparable to the findings by Locatelli et al. who showed that the standardized mortality in Switzerland was 8.6% higher in 2020 relative to 2019, likely reflecting the direct and indirect effects of the COVID-19 pandemic [19]. 

GI bleeding

Compared to 2019, our results showed that there was a decrease in hospitalizations in 2020 for nonvariceal GI bleeding, variceal upper GI bleeding, and lower GI bleeding, including diverticular bleeding. However, this trend was only statistically significant for non-variceal GI bleeding. We also observed a significant increase in hospitalization costs in 2020 for all types of GI bleeding. There was no statistically significant difference in terms of mortality between 2020 and 2019. For non-variceal upper GI bleeding, there was a trend of increased mortality in 2020 relative to 2019 for all months, except January and February. For lower GI bleeding including diverticular bleeding, there was a trend of increased mortality in 2020 relative to 2019 except in January, May, June, July, and October; however, there was no statistically significant difference in mortality overall. Merza et al. used data from the Centers for Disease Control and Prevention Wide-Ranging Online Data for Epidemiologic Research (CDC WONDER) database and found that the mortality rate increased from 3.3 per 100,000 to 4.3 per 100,000 among the population between 2012 and 2021, suggesting that the increase in mortality was attributable to COVID-19 [20]. The differences in our results might be given the difference in the years compared. Furthermore, their analysis used death certificate data, which might impact the results. 

Inflammatory bowel diseases and AP

We observed no significant difference in the rate of hospitalization for ulcerative colitis between 2019 and 2020. The peak number of admissions for ulcerative colitis occurred in January 2020, whereas the lowest number of admissions was in April 2020. Interestingly, we observed a significant increase in mortality and hospitalization costs among patients with ulcerative colitis in 2020 relative to 2019. This is an interesting finding deserving further investigation. There are multiple potential reasons for these observations such as immunosuppressive medications, severity of underlying inflammation, or comorbidities. It would be valuable to investigate the demographics at the highest risk of mortality among those with ulcerative colitis. However, unfortunately, the nature of the NIS dataset does not allow us to explore the use of biologics and other immunosuppressants that may independently increase the risk of COVID. For AP, we observed a significant decrease in hospitalization rates but a significant increase in hospitalization costs in 2020 relative to 2019. In 2020, the peak number of admissions for AP occurred in January, whereas the lowest number of admissions for all conditions occurred in April 2020. There was no statistically significant change in mortality in 2020 relative to 2019. These results are congruent with the work done by Ramsey et al., who illustrated decreased admissions and stable mortality rates for AP in 2020 relative to 2019 using a retrospective chart review from a single health center [8]. 

Diarrheal diseases

We investigated *Clostridium difficile*, viral gastroenteritis, and non-infectious gastroenteritis/colitis in this study. Compared to 2019, we observed a significant decrease in hospitalizations in 2020 for non-infectious colitis and viral gastroenteritis. There was also a trend of decrease in admissions for *Clostridium difficile*; however, it was not statistically significant. Given that diarrhea can be a symptom of COVID-19, we found it surprising that there was a drop in the admissions for viral gastroenteritis [21]. However, this might be secondary to misclassification or potential fear of presenting to the hospital during the pandemic [18]. Interestingly, mortality was significantly higher in 2020 relative to 2019 for noninfectious gastroenteritis/colitis; however, there was no difference in other diarrheal conditions. This mortality excess could potentially be attributed to COVID-19, especially since diarrhea could be a symptom of COVID-19. On the other hand, this observation could be the result of a delayed presentation given the significantly lower admissions for non-infectious gastroenteritis in 2020 (n=77,795) vs. 2019 (n=98,995). 

Diverticulitis

We observed a significant decrease in hospitalizations in 2020 for diverticulitis relative to 2019. In 2020, the peak number of admissions for diverticulitis occurred in January, which likely still represents the pre-pandemic era, whereas the lowest number of admissions was in April 2020, which corresponds to the initial phase of government lockdowns. There was no significant difference in mortality in 2020 relative to 2019; however, there was a trend of higher mortality in 2020 than in 2019 for all months except June, August, October, and November. Our results are similar to the work done by Hossain et al., who showed that fewer patients were admitted for acute diverticulitis during the pandemic in a single-center retrospective study [22].

COVID-19 mortality

As expected, we showed that older age increased the odds of death in a dose-dependent fashion and that patients over 75 years had the highest odds of death from COVID-19. There were no statistically significant effects of race, primary payer, or income on COVID-19 mortality in our sample. Our observation of higher mortality among patients in large and urban teaching hospitals is expected and we believe this reflects a referral bias, as patients in these centers tend to be sicker and more complex. We observed that the coexistence of hypertension, obesity, and a high Elixhauser comorbidity index ≥3 increased COVID-19 mortality among the cohort admitted with GI diseases. We also observed increased mortality in patients who were hospitalized for AP, variceal upper GI bleeding, ulcerative colitis, and acute cholangitis, who were also COVID-19 positive. A similar direction of significance was found in sensitivity analysis that accounted for time except for ulcerative colitis. A similar excess mortality of up to 7.7% was noted among patients admitted with both viral pneumonia and GI-related conditions in 2020 compared to 2019. These findings strongly suggest that some of these viral pneumonia cases might have represented COVID-19 and that the excess mortality is likely attributable to COVID-19 as well. 

Our study had some limitations. The observational design of our study and the use of administrative data limited our ability to establish direct causality, and we could only establish correlations [23]. Utilizing the NIS database prevented us from considering variables that might influence the results, such as the severity of the illness, vital signs, medication use, and laboratory test results of the patients in the study, as well as longitudinal tracking of the patients including post-discharge course [24]. However, using comorbidity indices such as the Elixhauser index, we were able to control for the underlying health conditions and possibly severity of disease by proxy. Furthermore, although the NIS database is quality-controlled, there is a possibility of coding errors given the reliance on International Classification of Diseases (ICD) codes, leading to the potential for misclassification bias particularly in the ascertainment of COVID-19, as the specific ICD-10 code was introduced in March 2020 and before this COVID-19 cases might have been coded as viral pneumonia [7,24]. Another limitation is that various states reacted differently to the pandemic, and thus, state-to-state variability was not captured in our analysis.

Despite these limitations, this study has several strengths. We utilized the largest inpatient sample in the United States which allows our results to have greater generalizability and a reduced chance of bias relative to single-center studies [23]. Furthermore, we were able to analyze month-to-month trends as well as year-to-year trends, including the pre-pandemic years, and our findings correlated to what we would expect with the phases of lockdowns and novel variants. These findings are important, as they contribute to our understanding of the impact of the COVID-19 pandemic and provide insights into the effects of potential future pandemics. Further research is necessary to validate these findings and investigate some of our unique findings such as the excess COVID-19 mortality observed among patients hospitalized with ulcerative colitis. In addition, more research is needed to explore conferred higher COVID-19 mortality and the impact of COVID vaccines and booster doses on inpatient GI outcomes, particularly at a population level.

## Conclusions

We used the NIS to analyze trends in GI-related hospitalizations during the COVID-19 pandemic. Overall, in the USA, acute GI-related hospitalizations decreased in 2020 but were linked to higher costs and increased all-cause mortality compared to the pre-pandemic period. Hospitalizations for non-variceal GI bleeding, diarrheal illness, and diverticulitis were statistically lower in 2020. Costs of hospitalization were significantly higher for GI bleeding. For unclear reasons, we observed a significant increase in mortality and hospitalization costs among patients with ulcerative colitis in 2020. We also observed increased mortality in COVID-19-positive patients hospitalized for AP, variceal, upper GI bleeding, ulcerative colitis, and acute cholangitis. Our results highlight important trends in acute GI-related hospitalizations including mortality trends on a national level in the USA. Further research is required to further delineate the underlying reasons for these observations. 
